# Precision‐Cut Bladder Slices: A Novel Model for the Study of Bladder Fibrosis and Potential Anti‐Fibrotic Agents

**DOI:** 10.1111/iju.70396

**Published:** 2026-03-07

**Authors:** Yutao Lu, Natasja Patricia Simonsen, Jens Christian Djurhuus, Lars Henning Olsen, Henricus Antonius Maria Mutsaers, Rikke Nørregaard

**Affiliations:** ^1^ Department of Clinical Medicine Aarhus University Aarhus Denmark; ^2^ Department of Renal Medicine Aarhus University Hospital Aarhus Denmark

**Keywords:** anti‐fibrotic compounds, bladder fibrosis, precision‐cut bladder slices, transforming growth factor‐β

## Abstract

**Objectives:**

Precision‐cut tissue slice culture is an innovative ex vivo approach for studying fibrosis pathogenesis. Here, we report for the first time the use of human precision‐cut bladder slices (PCBS) to investigate fibrotic changes and evaluate anti‐fibrotic compounds.

**Methods:**

Fresh bladder tissue was obtained from 16 patients undergoing surgery for non‐fibrotic conditions and 7 patients with documented bladder fibrosis. PCBS were cultured and stimulated with transforming growth factor β (TGF‐β) to induce fibrotic changes. Viability was assessed by ATP quantification. The anti‐fibrotic efficacy of pirfenidone (PFD), relaxin‐2 (RLN), bone morphogenetic protein 7 (BMP‐7), imatinib (IMA), and galunisertib (GAL) was evaluated. Fibrosis was quantified using qPCR analysis of collagen 1 (*COL1A1*), fibronectin (*FN1*), and cellular communication network factor 2 (*CCN2*) gene expression.

**Results:**

PCBS remained viable over 48 h, with stable ATP levels. TGF‐β stimulation significantly increased *COL1A1* and *CCN2* expression, confirming induction of a fibrotic response. Treatment with PFD, IMA, and GAL effectively attenuated TGF‐β‐induced upregulation of fibrosis markers. At baseline, PCBS derived from fibrotic bladders exhibited elevated *COL1A1* expression compared to non‐fibrotic tissue, while *FN* and *CCN2* levels remained unchanged. PFD treatment notably reduced *CCN2* expression in fibrotic PCBS.

**Conclusions:**

This study demonstrates that PCBS provide a viable and reproducible platform for modeling bladder fibrosis and screening anti‐fibrotic therapies. PFD, IMA, and GAL showed promising anti‐fibrotic effects, supporting further investigation into their therapeutic potential.

## 1 Introduction

Bladder fibrosis is a pathological consequence of various lower urinary tract disorders, including bladder outlet obstruction (BOO), spinal cord injury, posterior urethral valves, chronic radiation cystitis, and aging [1]. These conditions lead to excessive collagen deposition and extracellular matrix (ECM) remodeling, resulting in reduced bladder compliance and elevated intravesical pressures during the filling phase. Clinically, this can impair upper urinary tract drainage, contribute to renal dysfunction, and severely impact quality of life [2].

Although numerous in vitro and in vivo models [1] have been employed to study the mechanisms and potential therapies for bladder fibrosis, no effective treatment has been translated into clinical practice. One major limitation is the biological and anatomical divergence between experimental models and human tissue. For example, Gevaert et al. [3] demonstrated that interstitial cells in the human bladder display a myoid phenotype, in contrast to the fibroblast‐like phenotype observed in rodents, highlighting the importance of human‐relevant models.

The precision‐cut tissue slicing (PCTS) method has emerged as a powerful ex vivo tool in toxicology and pharmacology, particularly in liver, kidney, and lung research [4]. This technique preserves native tissue architecture and maintains essential cell–cell and cell–matrix interactions, making it suitable for modeling disease processes and evaluating drug responses over short‐term culture periods [5]. However, application of this approach to the bladder poses challenges due to its complex multilayered structure (urothelium, lamina propria, and detrusor muscle), which must be preserved during slicing and culturing.

To our knowledge, there are no published studies reporting precision‐cut bladder slices (PCBS) as an ex vivo model for investigating bladder fibrosis directly in human tissue. The primary aim of this study was to develop and validate a robust slicing and culture protocol for human bladder tissue that preserves its structural integrity and supports viability over 48 h.

Given the complex pathophysiology of bladder fibrosis and the prominent role of transforming growth factor‐β (TGF‐β) as a key profibrotic cytokine across organs, we utilized TGF‐β to induce fibrotic responses in PCBS. TGF‐β is secreted as a latent complex requiring cleavage and extracellular activation. In prior work, we observed upregulation of TGF‐β in murine bladders following 24 h of complete BOO [6]. Inhibition of the TGF‐β pathway, either genetically or pharmacologically, has shown promise in reducing ECM deposition and fibrosis in similar models [7].

Importantly, in addition to testing the fibrotic response in non‐fibrotic human tissue, we also generated PCBS from patients with clinically documented bladder fibrosis. This enabled us to explore the potential of anti‐fibrotic therapies not only for prevention but also for reversing or mitigating established fibrotic changes. Together, these models provide a human‐relevant, ex vivo platform for mechanistic studies and therapeutic screening in bladder fibrosis.

## 2 Methods

### 2.1 Tissue Collection

Bladder tissue samples were obtained from patients undergoing surgery for non‐fibrotic conditions without lower tract symptoms, including: bladder cancer, vesicoureteral reflux, and megaureter. Additionally, we included patients with clinically confirmed bladder fibrosis. The fibrosis cohort included patients that suffered from either neurogenic bladder or posterior urethral valves. Exclusion criteria included a warm ischemia time of more than 60 min or, for cancer patients, concomitant dysplasia in the bladder specimens. Patient demographics are presented in Table 1. Preliminary feasibility studies (Section 2.3) were performed using tissue samples from an anonymized patient cohort.

**TABLE 1:** Patient demographics.

	Non‐fibrotic	Fibrotic
No. (male/female)	16 (5/11)	7 (4/3)
Mean age (years)	61 ± 28 [1–86]	10 ± 6 [3–21]
Diagnosis (*n*)	Bladder cancer (12) Vesicoureteral reflux (2) Unilateral ureteral stenosis (1) Spinal muscular atrophy type II (1)	Neurogenic bladder (5) Posterior urethral valves (2)

*Note:* Values are presented as mean ± SD [range].

### 2.2 Preparation of PCBS

Full‐thickness bladder wall samples (1 × 1 cm) were excised and stored in University of Wisconsin (UW) solution at 4°C until slicing. In cancer patients, tissue was collected from macroscopically tumor‐free areas. The tissue was cut into 0.2 × 1 cm strips, embedded in 4% (w/v) agarose at 37°C, and cooled in a custom embedding chamber at 4°C to form a stable cylindrical block suitable for sectioning.

**FIGURE 1:**
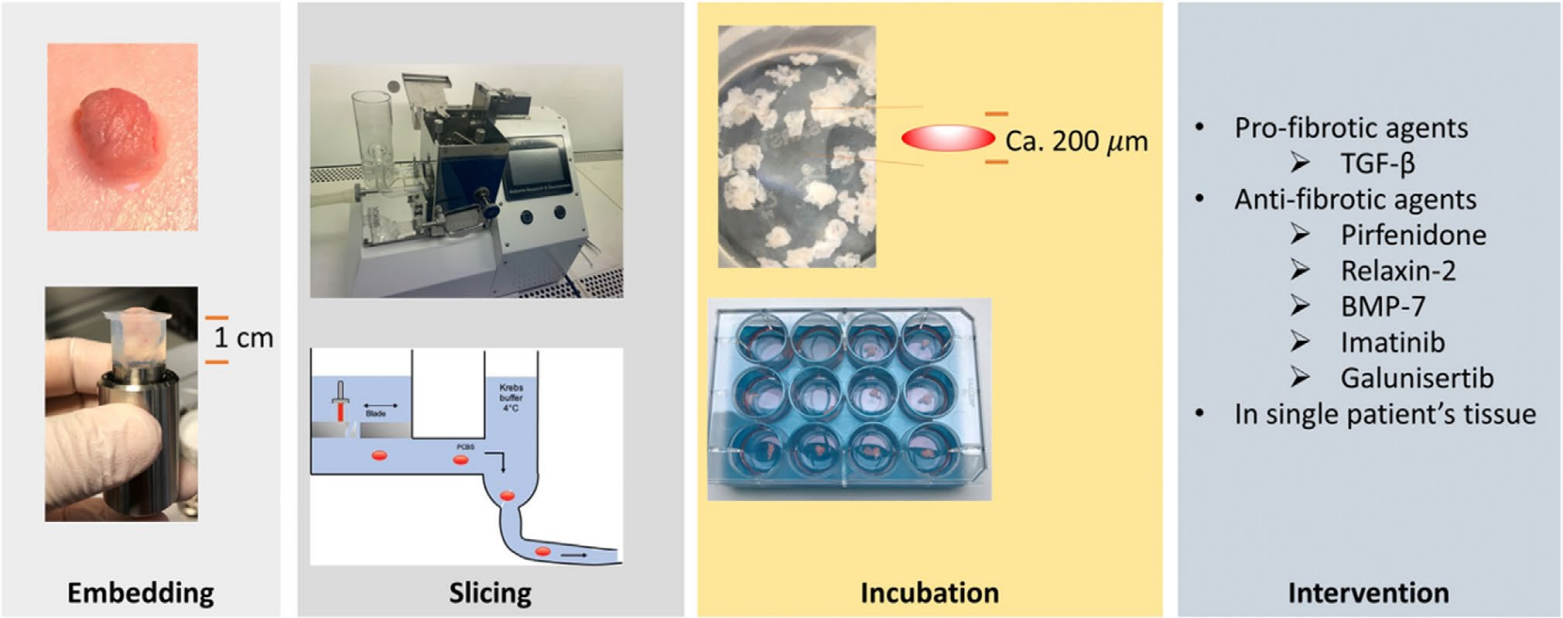
Workflow for preparation of precision‐cut bladder slices (PCBS). Bladder tissue was cut into 0.2 × 1 cm strips, embedded in 4% (w/v) agarose at 37°C, and cooled in a custom embedding chamber at 4°C to form a stable cylindrical block for sectioning. PCBS were then prepared using the Krumdieck tissue slicer, and each slice was incubated in optimized medium at 37°C with 80% O_2_ and 5% CO_2_ while being gently shaken (90 rpm). During incubation, PCBS were exposed to either TGF‐β (10 ng/mL) and/or various anti‐fibrotic compounds, depending on the experimental design.

Precision‐cut bladder slices (PCBS) were prepared in cold Krebs–Henseleit buffer using a Krumdieck tissue slicer (Alabama Research & Development, USA). Slices, with a thickness of circa 200 μm, were individually cultured in 600 μL of William's E medium supplemented with GlutaMAX (Life Technologies, Carlsbad, CA, USA), 10 μg/mL ciprofloxacin, and 2.7 g/L D‐(+)‐glucose (Sigma‐Aldrich, St. Louis, MO, USA). Cultures were maintained at 37°C in a humidified atmosphere (80% O_2_/5% CO_2_) on an orbital shaker moving at 90 rpm. Media was replaced every 24 h. The preparation process is illustrated in Figure 1.

### 2.3 Viability and Time Course Optimization

Due to the absence of prior studies using PCBS, a time course experiment was conducted to determine optimal incubation time with and without TGF‐β stimulation. Slices were harvested at 3, 24, 48, and 72 h, and viability was assessed at each time point using the ATP Colorimetric/Fluorometric Assay Kit (Sigma‐Aldrich), according to the manufacturer's instructions.

### 2.4 Anti‐Fibrotic Treatment Protocols

To evaluate the therapeutic potential of anti‐fibrotic compounds, PCBS were incubated with TGF‐β (10 ng/mL) to induce fibrotic gene expression, in the presence or absence of the following agents: pirfenidone (PFD, 2.5 mM, Sigma‐Aldrich, USA; *n* = 12), relaxin‐2 (RLN, 200 ng/mL, Sigma‐Aldrich, USA; *n* = 10), bone morphogenetic protein‐7 (BMP‐7, 500 ng/mL, PeproTech, USA; *n* = 8), imatinib (IMA, 25 μM, Sigma‐Aldrich, USA; *n* = 5), and galunisertib (GAL, 10 μM, Sigma‐Aldrich, USA; *n* = 4). We specifically chose compounds that are known to interact with the TGF‐β pathway. Experimental concentrations were based on prior studies with kidney slices as well as preliminary concentration‐response experiments [5].

### 2.5 Quantitative Polymerase Chain Reaction

Total RNA was isolated from PCBS using the Nucleospin RNA II Mini Kit (Macherey‐Nagel, Germany), following the manufacturer's protocol. RNA concentration was measured by spectrophotometry at 260 nm, and samples were stored at −80°C. Complementary DNA (cDNA) was synthesized from 0.5 μg RNA with the RevertAid First Strand synthesis kit (Thermo Scientific). Gene expression was quantified by real‐time PCR using gene‐specific primers (Table 2). To ensure reliable normalization, expression levels were normalized to two reference genes: glyceraldehyde‐3‐phosphate dehydrogenase (*GAPDH*) and TATA‐box binding protein (*TBP*). Relative expression was calculated using the 2^−ΔΔCt^ method, based on the geometric mean of *GAPDH* and *TBP* Ct values to account for potential variability in reference gene expression.

**TABLE 2:** Primer sequences.

Target gene	Forward primer	Reverse primer
*FN1*	5′‐CAGTGGGAGACCTCGAGAAG‐3′	5′‐GTCCCTCGGAACATCAGAAA‐3′
*COL1A1*	5′‐CCTGGATGCCATCAAAGTCT‐3′	5′‐AATCCATCGGTCATGCTCTC‐3′
*CCN2*	5′‐CTTGCGAAGCTGACCTGGAAGA‐3′	5′‐CCGTCGGTACATACTCCACAGA‐3′
*GAPDH*	5′‐ACCAGGGCTGCTTTTAACTCT‐3′	5′‐GGTGCCATGGAATTTGCC‐3′
*TBP*	5′‐TGTATCCACAGTGAATCTTGGTTG‐3′	5′‐GGTTCGTGGCTCTCTTATCCTC‐3′

### 2.6 Histology and Immunofluorescent Staining

To confirm structural preservation and ECM deposition, additional bladder slices were fixed in 4% paraformaldehyde for 1 h, rinsed in PBS, dehydrated in graded ethanol, and embedded in paraffin. Tissue sections (3 μm thick) were stained with Hematoxylin and Eosin (H&E) for general morphological evaluation and Masson's Trichrome for visualization of collagen deposition. Images were captured at 10× magnification from five randomly selected fields using the Olympus cellSens Imaging System (Olympus Corp., Tokyo, Japan). For quantitative analysis of fibrosis, Masson's Trichrome‐stained sections were analyzed using ImageJ software (NIH, Bethesda, MD, USA). The percentage of fibrotic tissue (blue‐stained regions) relative to the total tissue was calculated using color deconvolution and thresholding methods.

To visualize alpha‐smooth muscle actin (αSMA), tissue sections (3 μm thick) were deparaffinized, rehydrated, and then boiled in TEG‐buffer for 20 min. Afterwards, sections were left to cool and then incubated for 30 min in 50 mM NH_4_Cl. Next, the sections were incubated for 30 min in a blocking solution consisting of 0.1% BSA, 0.2% gelatine and 0.05% saponin in PBS. The sections were then incubated with a primary antibody against αSMA (1:1500, M0851, DAKO) overnight at 4°C, followed by incubation with an Alexa Fluor 488‐conjugated secondary antibody (1:400, A‐11001, Thermo Scientific) at RT for 30 min, and subsequently counterstained with DAPI. Slides were mounted with SlowFade Gold Antifade Mountant (S36936, Invitrogen), and images were obtained with an EVOS M5000 fluorescence microscope (Invitrogen) at 10× magnification.

### 2.7 Statistics

Data are presented as mean ± standard error of the mean (SEM), unless stated otherwise. Gene expression results are shown relative to pre‐incubation controls. Treatment response ratios were calculated based on mRNA expression as (TGF‐β + treatment/TGF‐β), with values < 1 indicating anti‐fibrotic activity. ECM quantifications are presented as medians with interquartile ranges (25th–75th percentiles). Group comparisons were performed using either a Mann–Whitney test, two‐tailed unpaired *t*‐test, or one‐way ANOVA with Fisher's LSD post hoc test. GraphPad Prism 10.1 (GraphPad Software, La Jolla, CA, USA) was used for all analyzes. A *p*‐value < 0.05 was considered statistically significant.

## 3 Results

### 3.1 Characterization of PCBS

**FIGURE 2:**
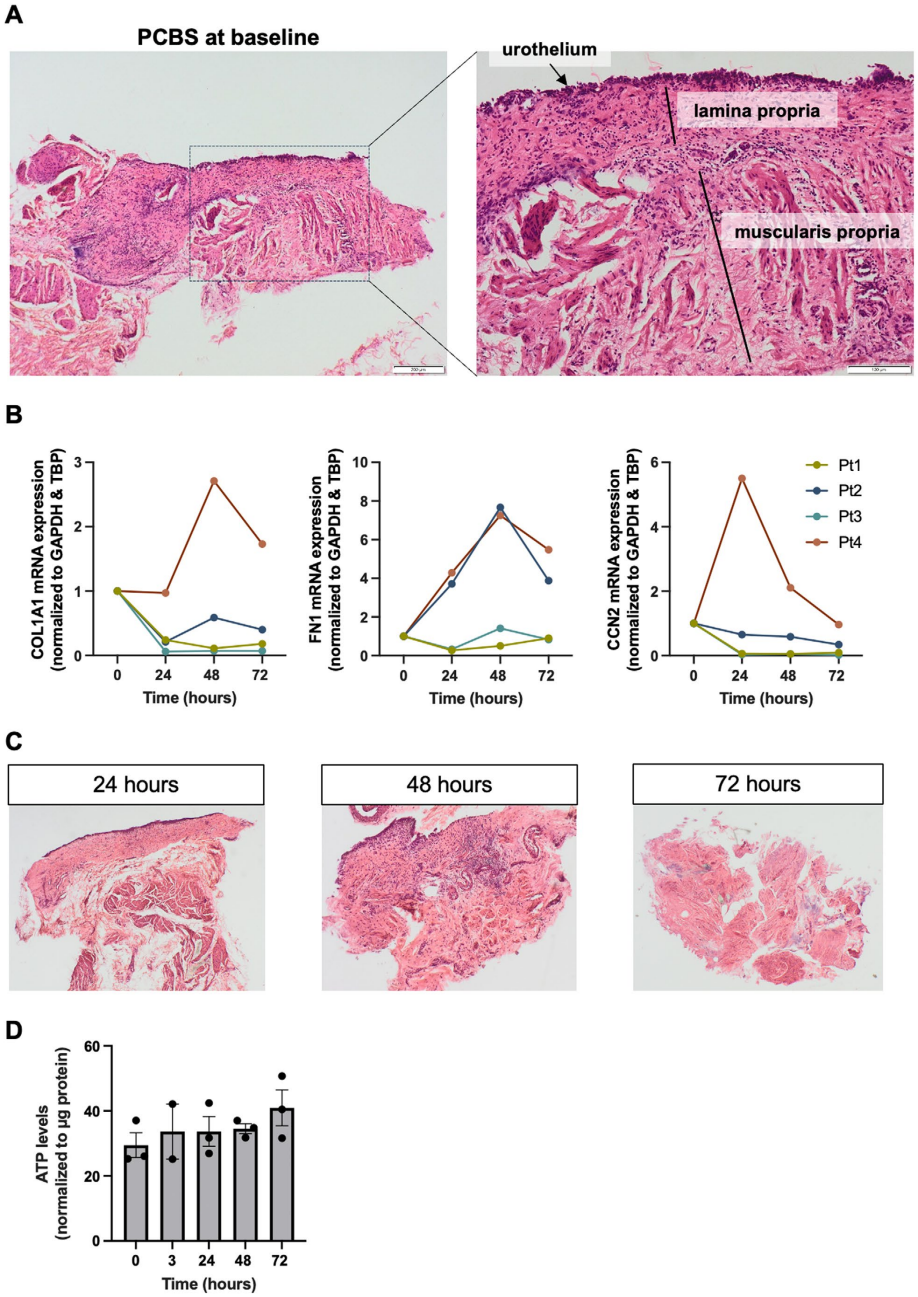
General morphology and viability of PCBS. (A) Histomorphology (H&E) of PCBS immediately after slicing. 10× magnification. (B) Gene expression of collagen 1 (*COL1A1*), fibronectin (*FN1*), and cellular communication network factor 2 (*CCN2*) in PCBS cultured for up to 72 h (*n* = 4). (C) Histomorphology (H&E) of PCBS after culture for up to 72 h. 10× magnification. (D) Viability of PCBS assessed by ATP content (*n* = 2–3). Data are shown as mean ± SEM.

After slicing, bladder histomorphology is well preserved in PCBS, with a clear distinction between the urothelium, lamina propria, and muscularis propria (Figure 2A). During culture, we observed that *COL1A1* and *CCN2* expression decreased in most donors, whereas *FN1* expression increased in the majority of cases (Figure 2B). In contrast, microscopy revealed that PCBS develop a fibrotic phenotype over time during culture (Figure 2C). After 24 h, normal tissue architecture is still maintained; however, by 48 h, the layered morphology became less defined, accompanied by an apparent increase in cellularity, which may indicate an accumulation of myofibroblasts. Furthermore, after 72 h, the urothelium and lamina propria are no longer discernible, and there is a marked increase in fibrotic matrix. Despite these fibrotic changes, ATP levels remained stable throughout the culture period, indicating that the slices remained viable (Figure 2D). Taken together, these observations suggest that PCBS are prone to spontaneous, that is, culture‐induced, fibrogenesis, a phenomenon that is also typically observed in slices from other organs [8, 9, 10].

### 3.2 TGF‐β‐Induced Fibrosis in PCBS

**FIGURE 3:**
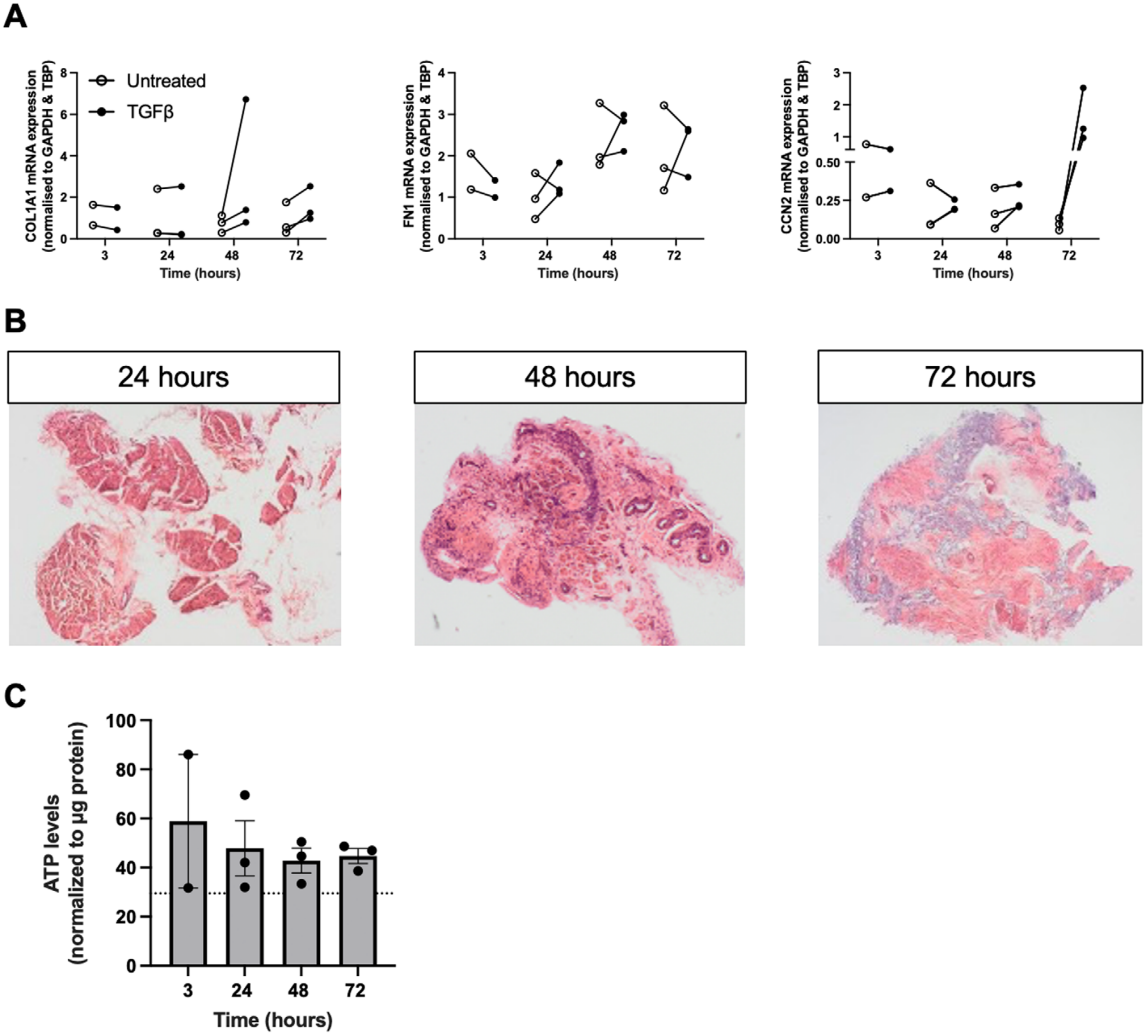
Effect of TGF‐β on PCBS. (A) Gene expression of collagen 1 (*COL1A1*), fibronectin (*FN1*), and cellular communication network factor 2 (*CCN2*) in PCBS treated with TGF‐β (10 ng/mL) for up to 72 h (*n* = 2–3). (B) Histomorphology (H&E) of TGF‐β–treated PCBS. 10× magnification. (C) Viability of TGF‐β‐treated PCBS assessed by ATP content (*n* = 2–3). Data are shown as mean ± SEM.

Next, we evaluated whether fibrogenesis could be further stimulated in PCBS by exposure to TGF‐β. As shown in Figure 3A, TGF‐β significantly upregulated *COL1A1* and *CCN2* expression after 48 and 72 h compared with untreated slices undergoing culture‐induced fibrosis, while its effect on *FN1* expression varied. In addition, TGF‐β markedly affected slice morphology (Figure 3B). At 24 h, the urothelium and lamina propria layers were already lost, and at both 48 and 72 h, this loss of tissue architecture was accompanied by an apparent increase in ECM deposition. Notably, TGF‐β did not affect slice viability, as demonstrated by stable ATP levels (Figure 3C). Taken together, these data indicate that PCBS are susceptible to TGF‐β‐induced fibrosis and can serve as a relevant model for screening potential anti‐fibrotic compounds.

### 3.3 PCBS as Drug Screening Platform

**FIGURE 4:**
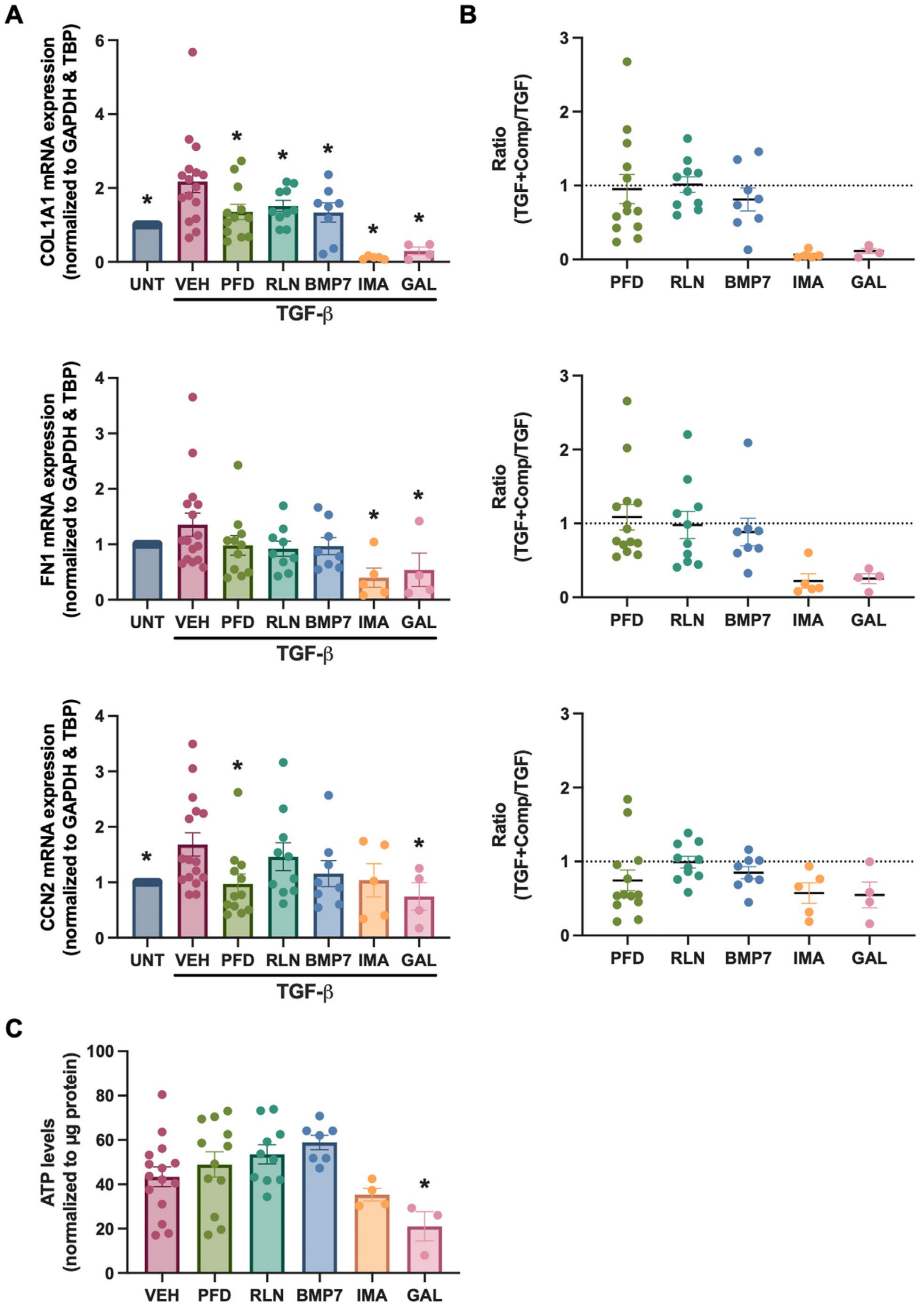
PCBS as a drug testing platform. (A) PCBS were incubated with TGF‐β (10 ng/mL) for 48 h in the presence or absence of the following agents: Pirfenidone (PFD, 2.5 mM; *n* = 12), relaxin‐2 (RLN, 200 ng/mL; *n* = 10), bone morphogenetic protein‐7 (BMP‐7, 500 ng/mL; *n* = 8), imatinib (IMA, 25 μM; *n* = 5), and galunisertib (GAL, 10 μM; *n* = 4). Gene expression of collagen 1 (*COL1A1*), fibronectin (*FN1*), and cellular communication network factor 2 (*CCN2*) was then evaluated by qPCR. (B) Ratio of treatment response; values < 1 indicate anti‐fibrotic activity. (C) Viability of PCBS following treatment, assessed by ATP content (*n* = 3–15). Data are shown as mean ± SEM. Statistical analysis was performed using one‐way ANOVA with Fisher's LSD post hoc test. *p < 0.05 compared to solvent control (VEH). UNT, untreated.

To further evaluate the potential of PCBS as a screening platform for anti‐fibrotic therapies, we evaluated the impact of pirfenidone, relaxin‐2, BMP‐7, imatinib, and galunisertib on TGF‐β‐induced fibrosis. As shown in Figure 4A,B, treatment with galunisertib significantly reduced the expression of *COL1A1*, *FN1*, and *CCN2* in TGF‐β‐exposed PCBS. Pirfenidone and imatinib both reduced *COL1A1* expression, along with *FN1* (imatinib) or *CCN2* (pirfenidone). On the other hand, relaxin‐2 and BMP‐7 solely reduced *COL1A1* expression. In addition, we observed that most compounds did not affect slice viability; however, imatinib and galunisertib did appear to slightly reduce ATP levels (Figure 4C).

### 3.4 Characterization of Fibrotic PCBS

**FIGURE 5:**
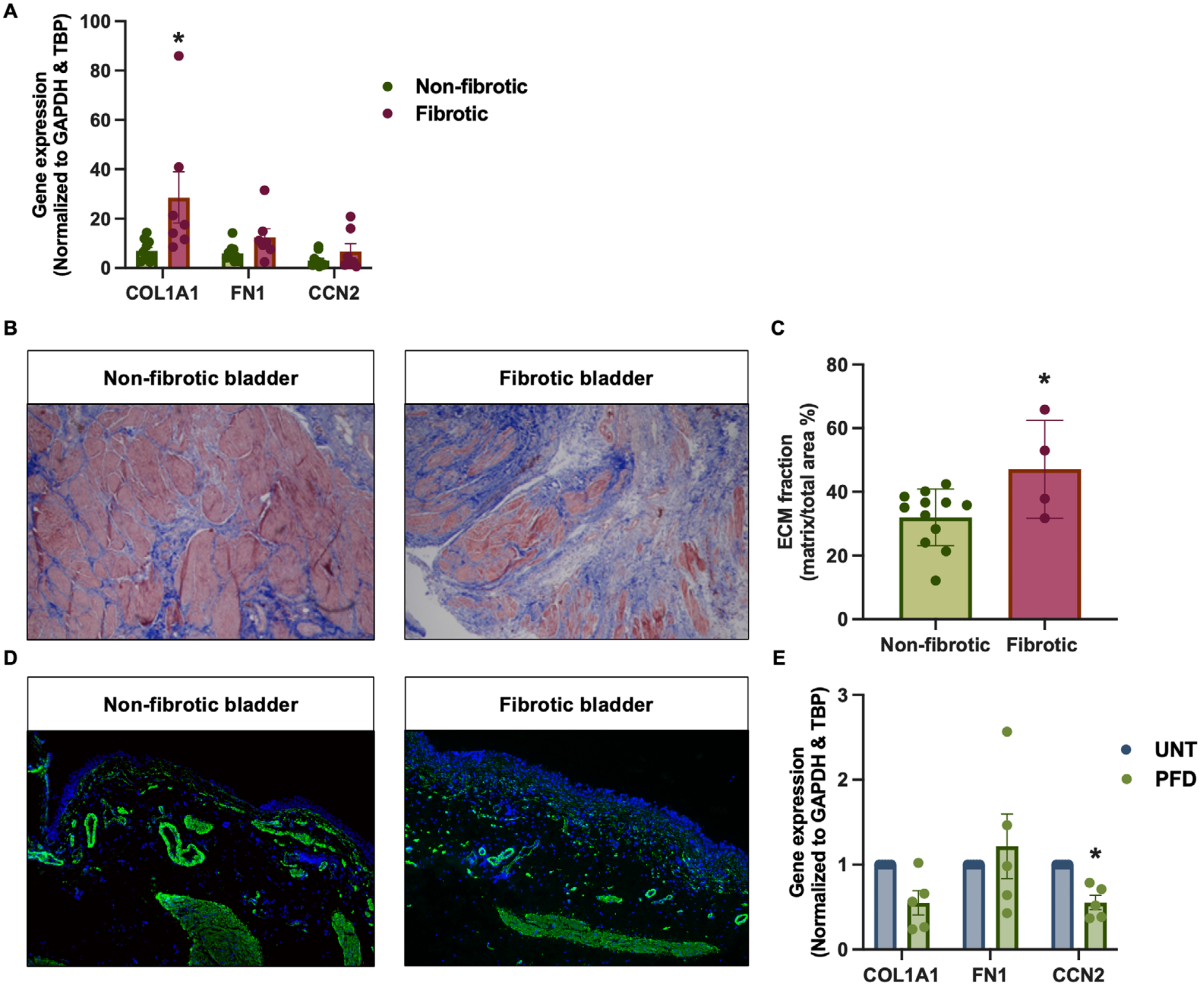
Fibrotic PCBS. (A) Baseline gene expression of collagen 1 (*COL1A1*), fibronectin (*FN1*), and cellular communication network factor 2 (*CCN2*) in non‐fibrotic and fibrotic PCBS (*n* = 7–10). (B) Masson's trichrome staining of non‐fibrotic and fibrotic PCBS. 10× magnification. (C) Quantification of Masson's trichrome staining, showing the percentage of fibrotic tissue (blue‐stained regions) relative to the total tissue (*n* = 4–12). (D) Representative image of immunolabeling for alpha‐smooth muscle Actin (αSMA; green) counterstained with DAPI (blue). 10× magnification. (E) Effect of pirfenidone (PFD, 2.5 mM) on *COL1A1*, *FN1*, and *CCN2* gene expression in fibrotic PCBS (*n* = 5). Data are shown as mean ± SEM. Statistical analysis was performed using either a Mann–Whitney test or two‐tailed unpaired *t*‐test. *p < 0.05 compared to either non‐fibrotic or untreated (UNT) slices.

Lastly, we tried to make slices from bladders with established fibrosis, as these could potentially be used to model late stages of the disease. As shown in Figure 5A, fibrotic slices had higher *COL1A1* expression as compared to PCBS prepared from non‐fibrotic tissue. In addition, we also observed that these slices had more fibrotic ECM (Figure 5B,C), conforming their fibrotic phenotype. In contrast, immunofluorescence staining of αSMA showed similar staining patterns in both non‐fibrotic and fibrotic slices and was primarily localized to the vessel walls and muscle layer (Figure 5D). Next, we evaluated the impact of pirfenidone on fibrotic PCBS. As shown in Figure 5E, pirfenidone significantly reduced *CCN2* expression following pirfenidone treatment, without significantly changing *COL1A1* and *FN1* expression.

## 4 Discussion

Precision‐cut tissue slices are a unique ex vivo model that preserves the native tissue architecture and cellular heterogeneity. This makes the model particularly well‐suited for studying complex multicellular disease processes, such as fibrosis. Here, we report for the first time the use of precision‐cut bladder slices (PCBS) for studying fibrosis and evaluating putative anti‐fibrotic therapies directly in viable human bladder tissue.

We demonstrated that PCBS remain viable in culture for up to 48 h. During this time, ATP levels remained constant and, more importantly, native bladder morphology was preserved. In addition, our data showed that PCBS spontaneously develop fibrosis during culture, probably due to the slicing procedure. This observation is in line with previous studies showing that human tissue slices prepared from liver, kidney, and intestine all develop fibrosis during culture [8, 9, 10]. We further demonstrated that, despite the presence of culture‐induced fibrosis, fibrogenesis could be further stimulated in human PCBS with TGF‐β. This is relevant since it is well documented that TGF‐β plays a critical role in bladder fibrosis, smooth muscle atrophy and reduced bladder compliance [11]. Our findings indicate that PCBS recapitulate in vivo bladder responses to TGF‐β, underscoring its physiological relevance as a model system.

Patient‐derived models play a central role in precision medicine, as they can be used to predict therapeutic responses and guide individualized treatment strategies. In recent years, patient‐derived organoids have emerged as a powerful tool in bladder cancer research because they faithfully recapitulate key aspects of parental tumor heterogeneity, including histological, molecular, and genetic features [12, 13]. They have been successfully applied to high‐throughput drug screening and shown to predict patient‐specific drug responses [13, 14, 15]. Despite these strengths, organoid cultures lack critical features of the native bladder microenvironment, including extracellular matrix, vasculature, stromal fibroblasts, and immune cells, which strongly influence disease progression and treatment outcomes [16]. PCBS address these limitations by preserving the multicellular complexity and spatial organization of the native bladder. This enables the study of cell–cell and cell–matrix interactions, as well as microenvironmental modulation of therapeutic responses. Importantly, PCBS can be generated directly from patient biopsies, allowing functional testing in the exact tissue context of the diseased bladder. Future studies should aim to evaluate if PCBS can be prepared from tumor tissue and can be used to evaluate drug responses and immunotherapy sensitivity. Such a patient‐specific ex vivo model would hold strong potential to guide personalized treatment decisions and predict clinical outcomes. Moreover, integration of functional readouts such as tissue compliance or contractility may further strengthen the model.

Despite these strengths, the PCBS model has certain limitations. Their short culture viability (typically 48–72 h) restricts experiments that need longer‐term monitoring of tissue responses, the limited number of slices obtainable from a single bladder biopsy constrains high‐throughput applications, and tissue availability depends on access to the necessary infrastructure for tissue procurement. Moreover, although PCBS preserve multicellular architecture, they lack systemic influences such as blood flow, circulating immune cell recruitment, and endocrine signaling, which can be critical for disease progression and drug metabolism. These limitations should be considered when designing experiments and interpreting findings.

Using PCBS as a drug testing platform, we demonstrated that pirfenidone was the most effective in reducing fibrosis in TGF‐β‐exposed slices without affecting ATP levels. This is in line with previous studies showing that pirfenidone attenuated bladder fibrosis in rats subjected to partial bladder outlet obstruction, as well as in a rat model of underactive bladder [17, 18]. Thus, pirfenidone might be a suitable drug candidate for the treatment of bladder fibrosis. It is worth noting that pirfenidone is already approved for the treatment of idiopathic pulmonary fibrosis [19], which might expedite its clinical application for bladder fibrosis.

In addition, both imatinib and galunisertib significantly reduced TGF‐β‐induced fibrosis in PCBS. The antifibrotic activity of imatinib has previously been demonstrated in human kidney slices [5], and galunisertib has been shown to exert antifibrotic effects in both human kidney and liver slices [5, 20]; however, to the best of our knowledge, this is the first report of their effects in a human model of bladder fibrosis. Several studies have reported beneficial effects of imatinib on bladder function in animal models of cystitis and spinal cord injury [21, 22, 23, 24, 25] as well as on pressure‐ and PDGF‐induced bladder smooth muscle cell hyperplasia [26]. Moreover, Qiao et al. demonstrated that imatinib sensitized bladder cancer cells (Ku80KD) to radiotherapy [27], and Zhuang and colleagues showed that galunisertib sensitized human urinary bladder cancer cells (T24) to chemotherapy [28]. Together, these findings suggest that both imatinib and galunisertib might hold potential as a therapeutic option for bladder fibrosis. However, we also observed a reduction in ATP levels following treatment with both compounds, which may indicate an impact on tissue viability. Therefore, further studies are required to determine the safety and clinical applicability of imatinib and galunisertib for the treatment of bladder fibrosis.

In contrast, neither BMP‐7 nor relaxin‐2 demonstrated anti‐fibrotic activity in human PCBS, despite prior reports of BMP‐7 mitigating fibrosis in multiple organs [29] and relaxin‐2 reducing bladder fibrosis in a mouse model of irradiation cystitis [30]. These discrepancies may reflect species‐specific differences in fibrotic pathways, reinforcing the importance of using human tissue models for preclinical drug evaluation.

Taken together, these findings highlight the potential of PCBS as a translational platform for drug testing in bladder fibrosis. This is especially relevant since there are currently no approved anti‐fibrotic therapies for patients with conditions such as radiation cystitis, bladder outlet obstruction, posterior urethral valves, or neurogenic bladder.

Our study has several limitations. First, we were only able to include seven patients with established bladder fibrosis, as this condition is relatively rare, particularly in the pediatric age group. Second, the included patients varied widely in age, which likely underlies the observed variability in individual drug responses, since aging is a well‐known contributor to fibrogenesis. Third, the cohort was too small to evaluate sex‐specific differences in fibrosis or drug responses. Fourth, the variation in sample sizes among treatment groups may complicate direct comparisons of drug efficacy.

In conclusion, we have successfully established human PCBS as a novel translational model for bladder fibrosis and demonstrated the applicability of this ex vivo model for drug screening. Pirfenidone, imatinib, and galunisertib showed promising anti‐fibrotic effects, warranting further research into their therapeutic potential for the treatment of bladder fibrosis.

## Author Contributions

**Yutao Lu:** investigation, formal analysis, visualization, writing – review and editing, writing – original draft. **Natasja Patricia Simonsen:** investigation, formal analysis, writing – review and editing. **Jens Christian Djurhuus:** conceptualization, supervision, project administration, writing – review and editing. **Lars Henning Olsen:** conceptualization, funding acquisition, supervision, project administration, writing – review and editing. **Henricus Antonius Maria Mutsaers:** formal analysis, project administration, visualization, writing – original draft, writing – review and editing, supervision. **Rikke Nørregaard:** conceptualization, investigation, formal analysis, funding acquisition, supervision, project administration, visualization, writing – original draft, writing – review and editing.

## Funding

This work was supported by Karen Elise Jensens Fond.

## Disclosure

Registry and the Registration No. of the Study/Trial: N/A.

Animal Studies: N/A.

## Ethics Statement

The use of human bladder tissue for the preparation of PCBS was approved by the Central Denmark Region Committees on Health Research Ethics (Journal no. 1‐10‐72‐243‐20) and registered in the internal records of the Central Denmark Region as the data controller.

## Consent

Written informed consent was obtained from all participants or their legal guardians.

## Conflicts of Interest

The authors declare no conflicts of interest.
